# Exercise and Glycemic Control: Focus on Redox Homeostasis and Redox-Sensitive Protein Signaling

**DOI:** 10.3389/fendo.2017.00087

**Published:** 2017-05-05

**Authors:** Lewan Parker, Christopher S. Shaw, Nigel K. Stepto, Itamar Levinger

**Affiliations:** ^1^Institute of Sport, Exercise and Active Living (ISEAL), College of Sport and Exercise Science, Victoria University, Melbourne, VIC, Australia; ^2^Institute for Physical Activity and Nutrition, School of Exercise and Nutrition Sciences, Deakin University, Geelong, VIC, Australia; ^3^Monash Centre for Health Research and Implementation, School of Public Health and Preventative Medicine, Monash University, Clayton, VIC, Australia; ^4^Australian Institute for Musculoskeletal Science (AIMSS), Victoria University and Western Health, St. Albans, VIC, Australia

**Keywords:** exercise, insulin signaling, stress kinase, glycemic control, oxidative stress, redox

## Abstract

Physical inactivity, excess energy consumption, and obesity are associated with elevated systemic oxidative stress and the sustained activation of redox-sensitive stress-activated protein kinase (SAPK) and mitogen-activated protein kinase signaling pathways. Sustained SAPK activation leads to aberrant insulin signaling, impaired glycemic control, and the development and progression of cardiometabolic disease. Paradoxically, acute exercise transiently increases oxidative stress and SAPK signaling, yet postexercise glycemic control and skeletal muscle function are enhanced. Furthermore, regular exercise leads to the upregulation of antioxidant defense, which likely assists in the mitigation of chronic oxidative stress-associated disease. In this review, we explore the complex spatiotemporal interplay between exercise, oxidative stress, and glycemic control, and highlight exercise-induced reactive oxygen species and redox-sensitive protein signaling as important regulators of glucose homeostasis.

## Introduction

Physical inactivity and excess adipose tissue are associated with the development of insulin resistance and type 2 diabetes mellitus (T2DM), which has reached epidemic proportions (1). Regular exercise can assist in the prevention and management of metabolic disease (2). Even a single session of exercise can improve glycemic control for up to 48 h postexercise (3–5). Improved glycemic control following acute and regular exercise occurs in part through improved insulin action and substrate metabolism in skeletal muscle (6, 7) by mechanisms that remain largely unknown. One potential mechanism may involve reactive oxygen species (ROS) and their paradoxical dual role in the pathophysiology of glucose homeostasis (8, 9). Considering that acute and chronic exercise training lead to alterations in oxidation–reduction (redox) homeostasis (10, 11), it is not surprising that redox biology has been proposed as a possible modulator of glycemic control and skeletal muscle adaptation to exercise (12–14). This review explores current evidence supporting exercise-induced ROS and skeletal muscle redox-sensitive protein signaling as important regulators of glucose homeostasis.

## Exercise and Glycemic Control

### Insulin-Stimulated Glucose Uptake

Glucose homeostasis is vital for organism survival and involves the complex interaction between intestinal glucose absorption, liver gluconeogenesis and glycogenolysis, and tissue glucose uptake (15). During conditions of elevated substrate availability, for example, a glucose load from a meal, elevated blood glucose is sensed by pancreatic β-cells resulting in the secretion of insulin to maintain glucose homeostasis (15). Under normal physiological conditions, insulin binds to the extracellular α-subunit of the insulin receptor promoting autophosphorylation of the transmembrane β-subunit on tyrosine residues 1158, 1162, and 1163 (16). Scaffolding proteins including Shc adapter protein isoforms, signal-regulatory protein family members, Gab-1, Cbl, adapter protein with a PH and SH2 domain, and insulin receptor substrates (IRS) are bound, and tyrosine residues phosphorylated to promote subsequent binding to phosphatidylinositol-3 kinase (PI3K) (17, 18). Activation of PI3K generates phosphatidylinositol (3,4,5)-trisphosphate (PIP_3_) that docks to and subsequently induces membrane translocation of the serine/threonine kinase Akt. PIP_3_ activation of phosphoinositide-dependent kinase-1 (PDK1) and the Rictor/mTOR complex 2 lead to dual phosphorylation of Akt on serine 473 and threonine residue 308 promoting subsequent activation of Akt kinase (19, 20). Increased Akt activity elicits phosphorylation of Akt substrate of 160 kDa (AS160; also known as TBC1D4) and TBC1D1 (21), promoting GTP loading and activation of Rab proteins releasing glucose transporter 4 (GLUT4) vesicles from intracellular compartments and promoting GLUT4 plasma membrane docking to facilitate glucose uptake (22–24).

**Figure 1:**
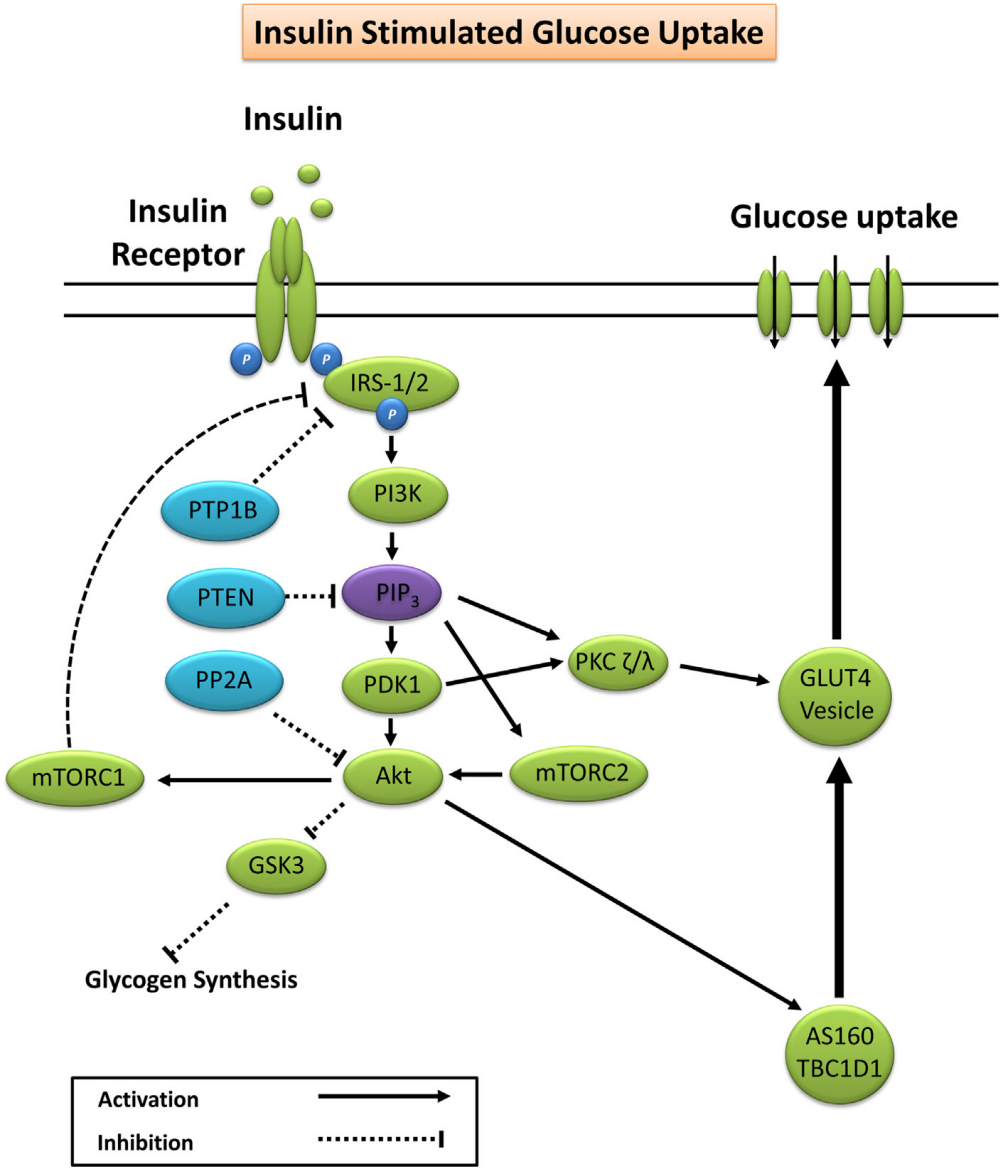
**Primary signaling pathways involved in insulin-stimulated glucose uptake**. Akt, protein kinase B; AS160, Akt substrate of 160 kDa; GLUT4, glucose transporter 4; GSK3, glycogen synthase kinase 3; IRS-1/2, insulin receptor substrates 1 and 2; mTORC1/2, mechanistic target of rapamycin complex 1/2; PDK1, phosphoinositide-dependent kinase-1; PI3K, phosphatidylinositol-3 kinase; PIP_3_, phosphatidylinositol (3,4,5)-trisphosphate; PKC, protein kinase C; PP2A, protein phosphatase 2; PTEN, phosphatase and tensin homolog; PTP1B, protein tyrosine phosphatase 1B.

Akt phosphorylation not only promotes GLUT4 translocation but also facilitates glycogen synthesis *via* inhibitory phosphorylation of glycogen synthase kinase 3 (GSK3) on Ser23 (GSK3α) and Ser9 (GSK3β) (25–27). PIP_3_ and PDK1 also activate atypical protein kinase C (PKC) isoforms ζ and λ, which are reported to facilitate GLUT4 vesicle trafficking and glucose uptake (28, 29). A summary of the canonical insulin signaling pathway is presented in Figure 1.

### Glucose Uptake during Exercise

**Figure 2:**
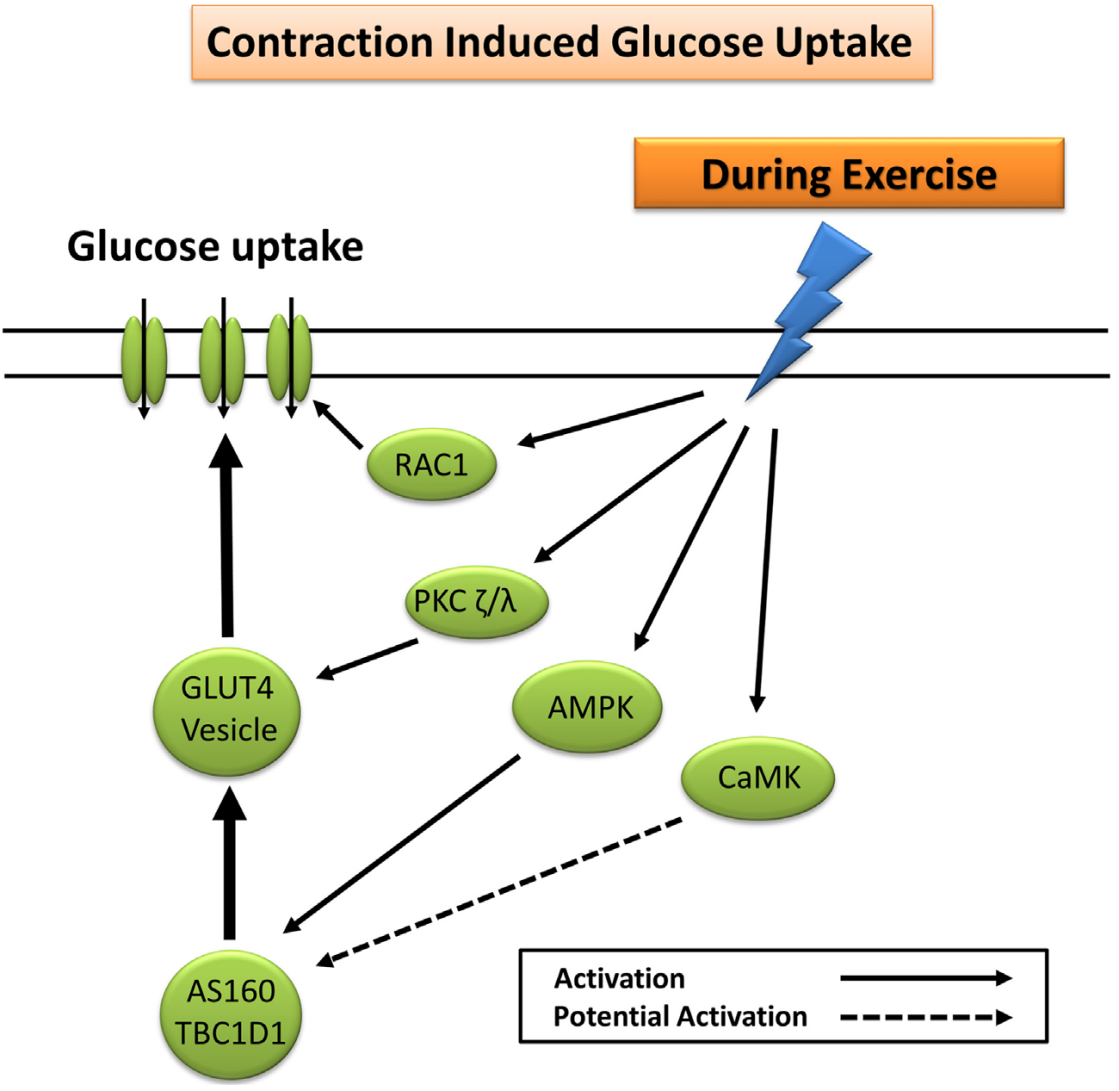
**Primary signaling pathways involved in contraction-induced glucose uptake**. AMPK, 5′ adenosine monophosphate-activated protein kinase; AS160, Akt substrate of 160 kDa; CaMK, Ca^2+^/calmodulin-dependent protein kinase; GLUT4, glucose transporter 4; PKC, protein kinase C; RAC1, ras-related C3 botulinum toxin substrate 1.

Glucose uptake during exercise occurs in an exercise intensity- and exercise duration-dependent manner, which depends largely on a combination of increased glucose delivery, glucose transport, and glucose metabolism (7). Increased trafficking of GLUT4 to the plasma membrane during exercise occurs largely through mechanisms independent of insulin (7). These include the cellular detection of changes in Ca^2+^ concentration (30, 31), changes in the energy status (ATP) of the cell (32–35), remodeling of the actin cytoskeleton *via* GTPase Rac1 (36), and fiber type-specific mediation of nitric oxide (NO) synthase (37). The primary protein signaling pathways include contraction-induced activation of calcium (Ca^2+^)/calmodulin-dependent kinase, atypical PKC, calcineurin, 5′ adenosine monophosphate-activated protein kinase (AMPK), Akt, and mitogen-activated protein kinases (12, 38). Exercise-induced AMPK, and to a lesser extent Ca^2+^ signaling pathways (30, 31), elicits GLUT4 translocation and subsequent glucose uptake through phosphorylation and inactivation of the convergent glucose uptake signaling proteins AS160 and TBC1D1 (21, 24, 39–42) (Figure 2).

### Postexercise Enhancement of Insulin Sensitivity

Glucose uptake during exercise is maintained in populations who are insulin resistant and/or have been diagnosed with type 2 diabetes (43). In contrast, basal and postexercise insulin-stimulated glucose uptake appears to be impaired and contribute to the development of chronic disease (8, 44, 45). Regular exercise in both healthy and clinical populations improves indices of glycemic control including glycated hemoglobin (HbA1c) and insulin sensitivity in a “dose”-dependent manner (duration and intensity) (2, 46). It is generally conceded that training-induced improvements in glycemic control lead to improved insulin action in part through the upregulation of key skeletal muscle glucose homeostasis regulatory proteins such as Akt1/2, AS160, AMPK, hexokinase 2, and importantly GLUT4 (6, 7). Improved insulin action may also occur through exercise-induced mitochondrial biogenesis and improved mitochondrial function in addition to the upregulation of antioxidant defenses that lead to improved redox homeostasis (6, 13).

In contrast to regular exercise, the transient enhancement of insulin sensitivity in the hours after acute exercise appear to occur independent of modifications to the insulin receptor, IRS1/2, PI3K, Akt, and/or GSK3 α/β proteins (3, 14, 47, 48). It has been reported that AS160 and TBC1D1, which converge downstream of insulin- and contraction-mediated glucose uptake signaling pathways, are associated with the postexercise enhancement of insulin sensitivity (14, 42, 49–53). Although decades of research have contributed to a greater understanding of exercise and glycemic control, the specific exercise-induced signaling mechanisms leading to the acute and long-term adaptations that favor enhanced glycemic control are less clear (3, 7). One potential mechanism may be through exercise-induced ROS and their capacity to act as second messengers for skeletal muscle cell signaling (13, 14, 54, 55).

## Redox Homeostasis

Biological organisms are constantly undergoing oxidation–reduction (redox) reactions to maintain a redox environment that is optimal for cellular signaling (56). Under certain circumstances, excess ROS production can lead to oxidative damage and/or modification of lipids, proteins, RNA, and DNA, leading to a redox state that is often referred to as oxidative stress (57). ROS production in a biological system occurs through numerous sources including radiation, environmental pollutants, chemotherapeutics, psychological stress (58), normal and abnormal cellular substrate metabolism (9, 59), and mechanical and physiological stress induced through exercise (9, 11). ROS considered to be of biological importance, which includes hydroxyl radical (OH), superoxide anion $\left({\text{O}}_{2}^{-}\right)$, NO, peroxyl radical, peroxynitrite, hypochlorous acid, hydrogen peroxide (H_2_O_2_), singlet oxygen, and ozone (57, 60). It should be noted that reactive nitrogen species and reactive sulfur species also constitute separate radical groups with independent biological functions (61, 62); however, their discussion lies beyond the scope of this review.

Reactive oxygen species are capable of direct and/or indirect oxidative modification to proteins (63). Sustained oxidation of proteins can result in disruptions in the normal functioning of the proteome including protein inactivation (64), modification of the protein side chains, fragmentation of peptide bonds (65), and structural unfolding and conformational changes (66). Likewise, ROS are implicated in oxidative damage to DNA, a process that ultimately results in strand breakage, DNA–protein crosslinks and base alterations, and defective DNA transcription and translation leading to the synthesis of less protein and/or defective protein (67–69). In addition to DNA, both messenger and ribosomal RNA are vulnerable to oxidative damage, which can lead to the disturbance of translational process and impairment of protein synthesis (69). ROS-induced damage to mRNA occurs primarily through the formation of highly reactive free radicals such as the OH (70) and appears to be selective and independent of the abundance of the mRNA species (69). Although RNA is highly susceptible to oxidative damage, considerably more so than DNA, protein, and lipids (69), to the authors knowledge, research has yet to investigate the effect of exercise-induced ROS production on RNA damage and the subsequent effects on protein synthesis and exercise adaption.

Lipids, especially polyunsaturated fatty acids, are susceptible to oxidative degradation, a process referred to as lipid peroxidation, which can result in a chain reaction leading to subsequent formation of peroxyl radicals and hydroperoxides (71). In addition to the direct cellular damage caused by ROS-induced lipid peroxidation, secondary products from lipid peroxidation such as malondialdehyde, propanal, hexanal, and the highly toxic 4-hydroxynonenal (4-HNE) can elicit signaling events that contribute to the development of cardiometabolic disease (72–75).

Disturbances in redox homeostasis can lead to perturbed redox signaling and aberrant cellular functioning (56). Therefore, organisms have evolved to encompass a complex and interconnected antioxidant defense system, which helps maintain redox homeostasis through the reduction of ROS and/or ROS intermediates, subsequent termination of ROS-mediated chain reactions, and/or through ROS-induced damage repair mechanisms (60, 76). These defenses include a number of redox-buffering enzymes, proteins, and scavengers, such as superoxide dismutase (SOD), catalase (CAT), glutathione peroxidase (GPx)/reductase, thioredoxin, peroxiredoxin, inducible nitric oxide synthase (iNOS), gamma-glutamylcysteine synthetase, redox effector factor 1, nuclear factor erythroid 2-related factor 2, antioxidant response element, Kelch-like ECH-associated protein 1, uric acid, lipoic acid, bilirubin, coenzyme Q10, vitamin C, vitamin E, and carotenoids (57, 60, 77–82).

## Oxidative Stress and Metabolic Health

Chronically elevated systemic oxidative stress is associated with over 100 pathological conditions including accelerated aging, cardiovascular disorders, insulin resistance, and T2DM (9, 57, 83). Considerable research has reported attenuated antioxidant defense and elevated basal oxidative stress in populations with chronic disease, often correlating with classical cardiometabolic risk factors such as increased circulating high-sensitivity C-reactive protein, greater waist-to-hip ratio, total cholesterol, triglycerides, and fasting blood glucose (84–90). As such, the measurement of basal systemic oxidative stress has been proposed as a marker for predicting the onset of a disease, assessing the progression of a disease, and evaluating the effect of pharmacological (e.g., antioxidant supplementation) and non-pharmacological (e.g., diet and exercise) therapies targeting oxidative stress-associated disease (81, 87, 91).

## Exercise-Induced Oxidative Stress

Acute exercise elicits a transient state of elevated ROS, which depending on the type of exercise, duration and intensity, and antioxidant capacity of the individual, can result in oxidative stress (11, 87, 92). In contrast to chronic oxidative stress, the transient increase in ROS and oxidative stress elicited by most types of exercise (i.e., non-extreme muscle damaging exercise) are reported to be beneficial and a necessary requirement for optimal cellular functioning and adaptation to physiological stress (79).

### Mechanisms for Exercise-Induced Oxidative Stress

**Figure 3:**
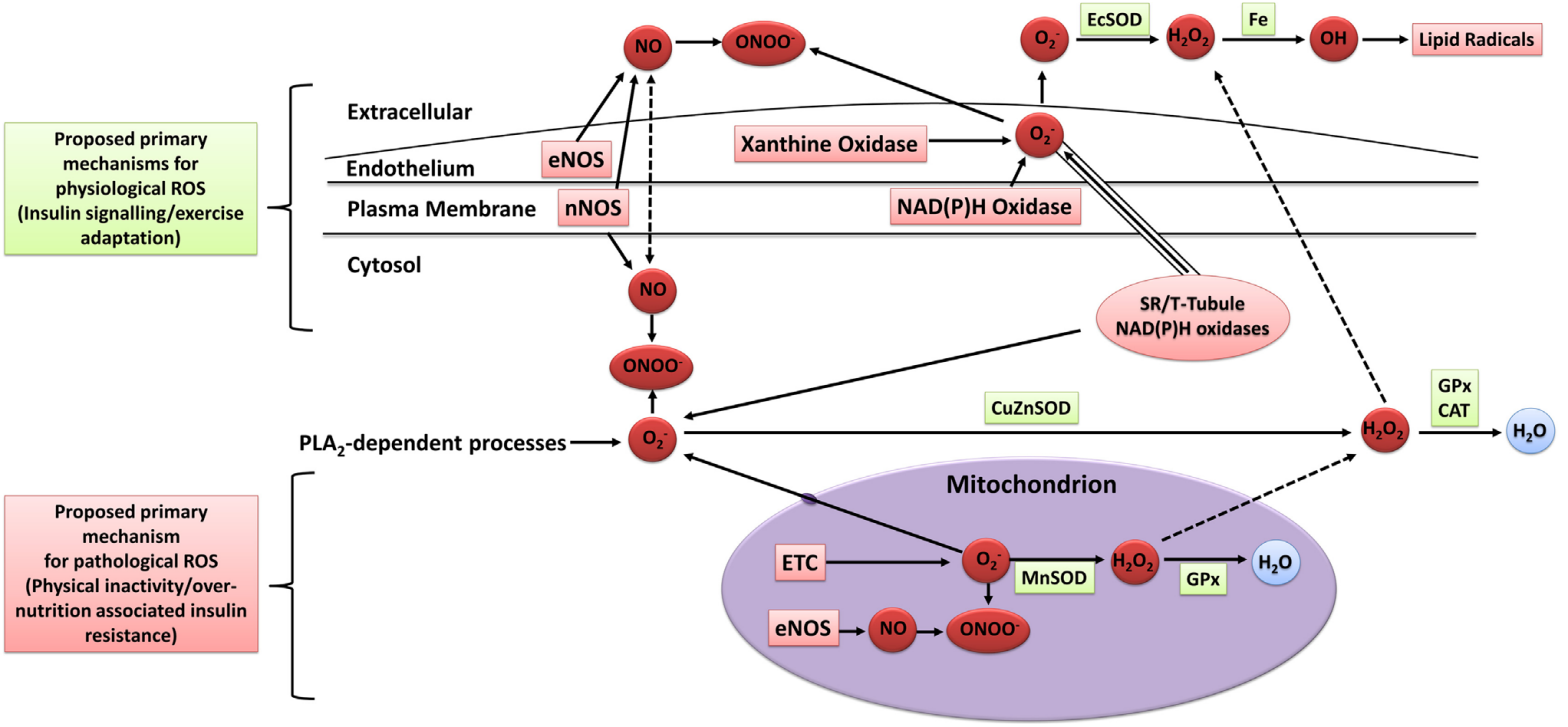
**Sources of ROS in skeletal muscle**. ETC, electron transport chain; eNOS, endothelial nitric oxide synthase; nNOS, neuronal nitric oxide synthase; NO, nitric oxide; ONOO^−^, peroxynitrite; OH, hydroxyl radical; ${\text{O}}_{2}^{-}$, superoxide; H_2_O_2_, hydrogen peroxide; H_2_O, water; EcSOD, extracellular superoxide dismutase; MnSOD, manganese superoxide dismutase; CuZuSOD, copper–zinc superoxide dismutase; GPx, glutathione peroxidase; CAT, catalase; PLA_2_, phospholipase A2; Fe, iron; ROS, reactive oxygen species. Adapted from the study by Powers and Jackson (93) with permission.

The mechanisms of intracellular and extracellular ROS generation in skeletal muscle during exercise are reviewed in detail elsewhere (93–95). In brief, the primary mechanisms are suggested to include NADPH oxidase (96, 97), xanthine oxidase (98), NO synthase (99), and arachidonic acid release from cell membranes by phospholipase A2 (100), whereas mitochondrial electron leak is suggested to contribute only marginally during muscular contraction (101) (Figure 3). Other mechanisms that may contribute to elevated skeletal muscle and/or plasma oxidative stress include the oxidation of catecholamine (102), lactate accumulation (103, 104), elevated core body temperature (105), hemoglobin and myoglobin-mediated autooxidation (106–108), and postexercise inflammatory and phagocytic responses including ischemic reperfusion, cytokine secretion, and respiratory burst (109–111).

Although plasma oxidative stress is commonly measured as an indicator of exercise-induced oxidative stress, the exact sources of systemic oxidative stress following skeletal muscle contraction are not well understood. Nevertheless, due to the large proportion of body mass that is constituted by skeletal muscle, it is proposed that skeletal muscle fibers, vascular cells, endothelial cells, and/or blood cells residing within skeletal tissue are the main contributors of both the exercise-induced local and systemic oxidative stress (95). *Ex vivo* skeletal muscle contraction studies have established the potential for skeletal muscle to elicit systemic oxidative stress (95, 112, 113). The specific cell types that contribute to skeletal muscle ROS production likely include vascular smooth muscle cells, endothelial cells, fibroblasts, erythrocytes, and white blood cells, with skeletal muscle fibers suggested to play the biggest role in the generation of extracellular ROS during and after exercise (95, 114, 115). Other tissues such as the heart, liver, and lungs may also contribute to the systemic increase in oxidative stress following acute exercise, but likely to a lesser degree (95).

### Exercise-Induced Oxidative Stress and Metabolic Health

To date, the literature is equivocal in regards to the effect of acute exercise on biomarkers of oxidative stress and antioxidant activity (11). Inconsistencies in the literature likely result from variations in dietary intake, training status, exercise intensity (5, 11, 92, 116, 117), exercise duration (11, 118, 119), exercise mode (11, 119), tissues sampled (119), sampling time points (119, 120), as well as the variety and volatility of the biochemical assays used (121). Nevertheless, the general consensus is that acute exercise elicits a transient increase in systemic and localized oxidative stress and antioxidant defense, which, depending on the intensity and mode of exercise, can be detected for up to 4 days after exercise (11, 116, 122).

**Figure 4:**
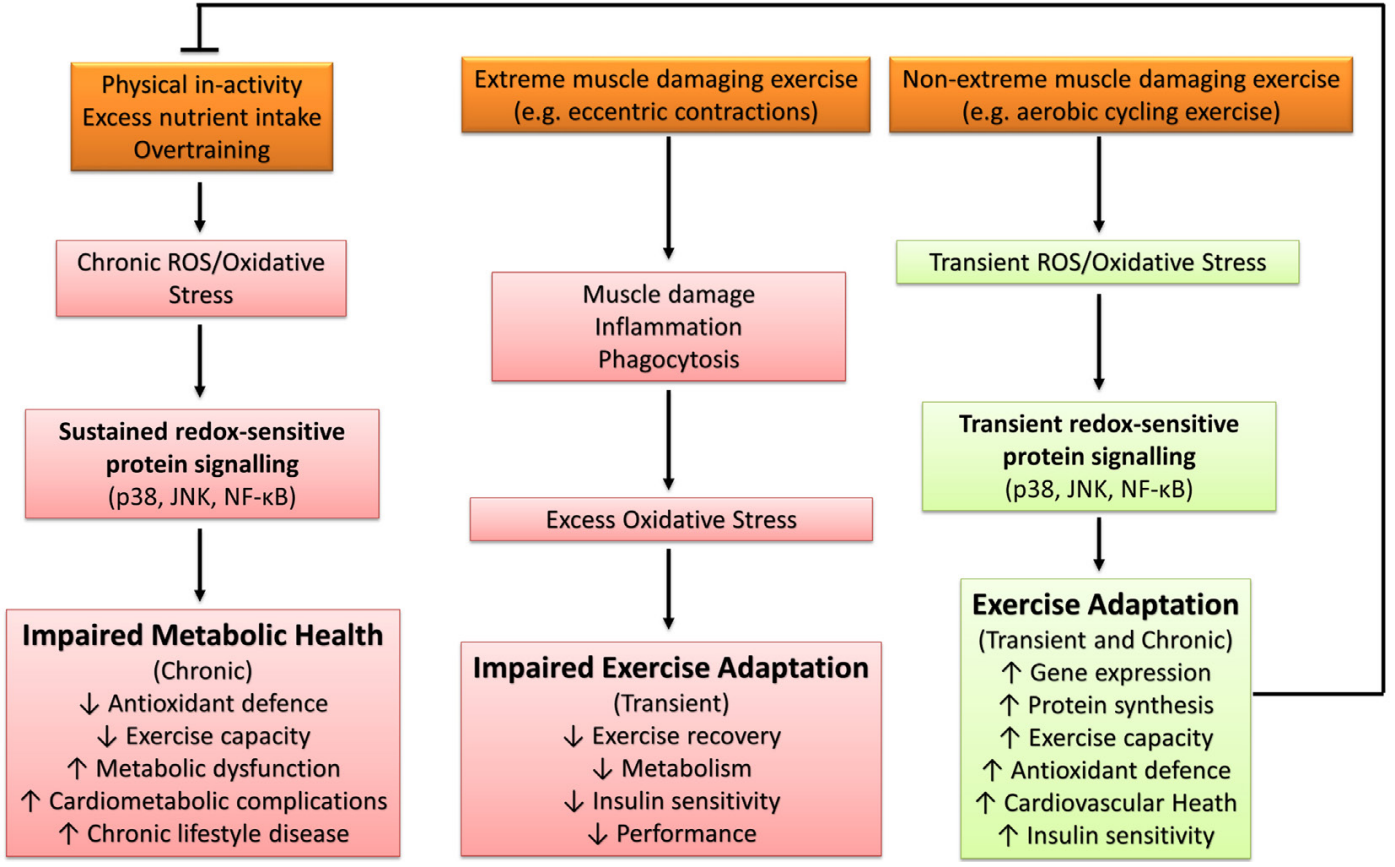
**The influence of oxidative stress in health and disease**. p38, p38 mitogen-activated protein kinases; JNK, c-Jun *N*-terminal kinases; NF-κB, nuclear factor kappa-light-chain-enhancer of activated B cells.

Excessive ROS production and/or oxidative stress induced through severe or extreme exercise regimes (e.g., ultraendurance events) in humans is associated with cellular disturbances promoting muscular fatigue (94, 123), aberrant upregulation of endogenous antioxidant defenses (124, 125), and impaired cognitive function (126). Similarly, impaired exercise tolerance and physiological responses have been documented in murine animal models (127). For example, Aoi et al. (128) reported that muscle damaging exercise in mice induced through downhill running increased skeletal muscle oxidative stress [thiobarbituric acid reactive substances (TBARS)] and resulted in 4-HNE-mediated impairment of the canonical insulin protein signaling pathway and decreased insulin-stimulated glucose uptake 24 h after exercise. Thus, under certain conditions, exercise-induced oxidative stress has the potential to elicit a deleterious redox environment conducive to impaired exercise capacity and health (Figure 4).

The pathological effects of exercise-induced oxidative stress likely stem from secondary muscle damage leading to phagocytic infiltration into skeletal muscle (129) and subsequent generation of ROS (130, 131). In support, Nikolaidis et al. (122) reported that muscle damaging exercise (75 lengthening knee flexions) significantly increased serum oxidative stress (TBARS, oxidized GSH, and protein carbonyls) and serum antioxidant defense (CAT activity, uric acid, bilirubin, and total antioxidant capacity), which lasted for up to 4 days after exercise. When a second identical bout of exercise was performed 3 weeks later, indices of muscle damage were lower, including improved isometric torque, which coincided with attenuation of the postexercise systemic redox response (122). Thus, the acute exercise-induced oxidative stress impairment of exercise performance, recovery, and metabolic health appears to occur independently from the transient and immediate increase in oxidative stress measured during and after exercise and is likely attenuated with subsequent exercise-induced oxidative stress insults (e.g., exercise training).

The majority of literature supports the idea that transient ROS production and/or oxidative stress elicited through regular exercise regimes (e.g., accustomed and/or non-extreme muscle damaging exercise) is beneficial and a necessary requirement for optimal physiological functioning and adaptation to physiological stress (79). Samjoo et al. (132) reported that 12 weeks of endurance training (2–3 sessions per week of 30–60 min cycling at 50–70% VO_2peak_) in obese and sedentary men decreased basal skeletal muscle and urinary markers of oxidative stress (4-HNE, protein carbonyls, and 8-isoprostane), increased basal skeletal muscle MnSOD protein abundance, and improved indices of glycemic control. Thus, repetitive sessions of exercise-induced ROS (i.e., exercise training) can improve metabolic health through the upregulation of endogenous antioxidant defense and attenuation of basal chronic oxidative stress. Further support for the beneficial effect of exercise-induced ROS can be found in human and animal studies that have reported antioxidant compounds to impair exercise capacity (133, 134), adaptive gene expression and protein synthesis (133, 135–138), upregulation of antioxidant defense (10, 13, 133, 136, 139, 140), cardiovascular health (141, 142), skeletal muscle inflammatory response and repair capabilities (134, 139), and insulin sensitivity (13, 55, 143, 144). Not all studies have reported the blunting of the aforementioned exercise-mediated adaptations (145–149), with some reports indicating enhanced exercise-induced adaptation with antioxidant supplementation (150, 151). An overview of the diverse role of oxidative stress in metabolic health is presented in Figure 4.

## Stress-Activated Protein Kinase (SAPK) and Mitogen-Activated Protein Kinase (MAPK) Signaling

Stress-activated protein kinase and MAPK signaling pathways include, but are not limited to, p38 MAPK (p38 MAPK), c-Jun *N*-terminal kinases (JNK), nuclear factor kappa-light-chain-enhancer of activated B cells (NF-κB), and PKC (152, 153). For the purpose of this review, both MAPK and SAPK are collectively referred to as SAPK.

Stress-activated protein kinase signaling pathways are associated with cellular proliferation, differentiation, survival, and cell death. Uncontrolled or sustained activation of SAPK signaling pathways are associated with the development and progression of cancer, neurodegenerative, and cardiometabolic disease (8, 57, 154). In contrast, controlled and/or transient SAPK activation is required for normal physiological functioning and reported to mediate many of the adaptations and health benefits received from regular exercise (12, 152).

Stress-activated protein kinase pathways are activated through numerous stimuli involving hormones, growth factors, cytokines, agents acting through G protein-coupled receptors, transforming growth factors, pathogens and danger-associated molecular patterns, and physical and chemical stresses (153, 155, 156). Relevant to the current review, however, is the inherent capacity of ROS to both directly and indirectly activate SAPK signaling pathways in skeletal muscle (157–161).

### ROS-Induced SAPK Signaling

The direct oxidation of proteins on cysteine residues by ROS act as biological “switches” turning on the catalytic properties of numerous proteins and enzymes (162). Cysteine thiol oxidation produces sulfenic acids, which form irreversible oxidation products or, in many cases, react to form reversible disulfide and sulfenamide bonds. These bonds can later be reduced *via* enzymes or compounds such as thioredoxin and glutathione, acting as an “off switch” and inhibiting protein function and enzymatic activity (163, 164). ROS-induced SAPK signaling can occur through reversible oxidative modification processes that involve MAPK kinase kinases (MAP3K/MAP2Ks) (165) and oxidative inactivation of thioredoxin (166, 167) and MAPK phosphatases (168–171). In addition, SAPK activation can occur through ROS-induced inactivation of glutathione S-transferases (172), tyrosine phosphorylation of protein kinase D (173), tyrosine, and serine phosphorylation of upstream targets such as the nuclear factor of kappa light polypeptide gene enhancer in β-cells inhibitor alpha (174) and the interaction with growth factor and cytokine receptors (163, 175). Crosstalk also exists between SAPK signaling pathways, with activation of one pathway (e.g., JNK and p38 MAPK) often interacting with and activating other pathways (e.g., NF-κB) (176). Irrespective of the mechanisms, considerable research has reported increased SAPK signaling under conditions of elevated ROS production (135, 157–160).

### Exercise-Induced SAPK Signaling

The mechanical and physiological stresses elicited by acute exercise are potent stimuli for the transient activation of SAPK signaling in human skeletal muscle in part through increased ROS production (12). Exercise-induced SAPK signaling activate important skeletal muscle transcription factors and coactivators, which include peroxisome proliferator-activated receptor gamma coactivator 1-alpha (PGC-1α), activating transcription factor 2, myocyte-enhancing factor 2, c-jun, c-fos, p53, and Elk-1 (12, 135, 177–185). Exercise-induced SAPK signaling is also associated with increased gene expression and the upregulation of antioxidant defenses such as SOD, iNOS, gamma-glutamylcysteine synthetase, GPx, and CAT (12, 135, 137, 161, 185–187).

Evidence supporting a role for exercise-induced ROS and SAPK signaling in exercise adaptation is primarily derived from research manipulating the redox environment to attenuate or enhance the exercise-induced ROS and protein signaling response. Henriquez-Olguin et al. (161) reported that inhibition of the ROS-producing enzyme complex NADPH oxidase 2 in rats attenuates the exercise-induced skeletal muscle phosphorylation of p38 MAPK and NF-κB p65 and gene expression of MnSOD, GPx, citrate synthase (CS), and mitochondrial transcription factor A (mtTFA). Similar findings have also been published using ROS inhibitors (e.g., antioxidant supplementation) in animals (10, 135, 188). Strobel et al. (189) reported that increased exercise-induced oxidative stress *via* skeletal muscle glutathione depletion in rats resulted in greater PGC-1α gene expression. In humans, antioxidant supplementation attenuates exercise-induced activation of p38 MAPK, NF-κB p65 and JNK protein signaling, and gene expression of SOD isoforms in skeletal muscle (10, 134, 137). Chronic inhibition of exercise-induced oxidative stress also impairs the training-induced upregulation of PGC-1α, nuclear respiratory factor (NRF)-1, and mtTFA in rats (135).

It is important to note that not all studies have reported an association between increased redox-sensitive protein kinase signaling and exercise adaptation. Wadley et al. (190) reported similar PGC-1α, NRF-2, and SOD gene expression after exercise in rats with allopurinol treatment, a xanthine oxidase inhibitor, despite decreased p38 MAPK phosphorylation and mtTFA gene expression. In addition, chronic allopurinol treatment was reported to have no effect on the training-induced upregulation of PGC-1α, mtTFA, cytochrome c, CS, and β-hydroxyacyl-CoA dehydrogenase (190). In humans, Morrison et al. (140) reported vitamin C and E supplementation to have little effect on exercise-induced gene expression of PGC-1α, mtTFA, and PGC-related coactivator, or training-induced improvements in VO_2peak_, CS activity, and expression of cytochrome oxidase subunit 4. However, SOD activity and protein abundance of SOD and mtTFA were attenuated by vitamin C and E supplementation (140). A summary of key findings from research investigating redox manipulation, exercise, and SAPK signaling are summarized in Table 1.

**Table 1:** **Summary of key findings from research investigating the effect of redox state manipulation on acute exercise-induced protein signaling and molecular markers of skeletal muscle adaptation**.

Reference	Participants	Exercise	Redox manipulation	Time point	SAPK signaling^a^	Markers of skeletal muscle adaptation^a^
Gomez-Cabrera et al. (10)	25 adults Trained Healthy	Marathon	Allopurinol (*n* = 14) Placebo (*n* = 11)	Postex.	Placebo only: ↑ lymphocyte NF-κB p50 activity	

Gomez-Cabrera et al. (188)	15 male Wistar rats	Exhaustive treadmill exercise	Rest (*n* = 5) Exercise (*n* = 5) Exercise + allopurinol (*n* = 5)	Postex.	Placebo only: ↑ p-p38 MAPK, NF-κB activity	Placebo only: ↑ MnSOD, iNOS, and eNOS mRNA

Henriquez-Olguin et al. (161)	20 male BalbC mice	Swimming exercise	Apocynin (*n* = 10) Vehicle (*n* = 10)	Postex.	Apocynin: attenuated p-p38 MAPK and p-NF-κB p65	Apocynin: attenuated MnSOD, GPX, CS, and mtTFA mRNA

Kang et al. (135)	18 female Sprague-Dawley rats	Exhaustive treadmill exercise	Allopurinol (*n* = 9) Vehicle (*n* = 9)	Postex.	Allopurinol: attenuated p–p38 MAPK, p-IκBα, NF-κB DNA binding	Allopurinol: attenuated PGC-1α, p-CREB, NRF-1, mtTFA content

Michailidis et al. (134)	10 young males Active Healthy	300 unilateral eccentric leg repetitions	Crossover: *N*–acetylcysteine and placebo	2 h postex.	Both: ↑ p-p38 MAPK	Both: ↑ p-Akt^Ser473^, p–p70S6K^Thr389^ and p-rpS6. NC MyoD. Muscle function impaired (mean torque)
					NC p-NF-κB p65	
				2 days postex.	NAC: greater p-p38 MAPK Attenuated p-NF-κB p65	Both: ↑ p-Akt^Ser473^. NC MyoD. NAC: attenuated mTOR^Ser2448^, p-p70S6K^Thr389^ and p-rpS6. Muscle function impaired.
				8 days postex.	Both: NC p-NF-κB p65 NAC: Attenuated p-p38 MAPK	NAC: attenuated p-Akt^Ser473^, mTOR^Ser2448^, p-p70S6K^Thr389^, p-rpS6, and MyoD Placebo only: muscle function completely recovered

Petersen et al. (137)	8 young males Trained Healthy	45 min at 71% VO_2peak_ followed by 92% VO_2peak_ to fatigue	Crossover: *N*–acetylcysteine and Saline infusion	Postex. (45 min at 71% VO_2peak_)	Both: ↑ p-p38 MAPK, ↓ IκBα. NC p-NF-κB p65. NAC: attenuated p-JNK	Both: ↑ PGC-1 α mRNA. NAC: attenuated MnSOD mRNA
				Postex. (fatigue)	NAC: attenuated p-JNK, ↓ p-NF-κB p65.	Both: NC PGC-1 α mRNA and MnSOD mRNA

Strobel et al. (189)	Male Wistar rats	Exhaustive treadmill exercise	DEM and controls	Postex. 4 h postex.	Both ↑ p-p38 MAPK Not measured	Not measured Both: NC NRF-2 DEM: greater ↑ PGC-1α mRNA. Attenuated GPx mRNA

Trewin et al. (55)	7 young adults Active Healthy	55 min at 65% VO_2peak_ followed by 5 min at 85% VO_2peak_	Crossover: *N*–acetylcysteine and Saline infusion	Postex.	NAC: ↓ p-p38 MAPK	Both: ↑ p-p70S6K^Thr389^ and p-rpS6

Wadley et al. (190)	Male Sprague-Dawley rats	Treadmill exercise	Allopurinol or placebo	Postex. 4 h postex.	Allopurinol: attenuated p–p38 MAPK Not measured	Not measured Both: ↑ mtTFA, NRF-2, PGC-1α, GLUT4, MnSOD, and EcSOD mRNA Allopurinol: attenuated mtTFA mRNA

*^a^Molecular response in skeletal muscle unless otherwise noted*.

*NC, no change compared to baseline or control; ↑, significant increase compared to baseline or control; ↓, significant decrease compared to baseline or control; iNOS, inducible nitric oxide synthase; eNOS, endothelial nitric oxide synthase; CS, citrate synthase; mtTFA, mitochondrial transcription factor A; NRF-1/2, nuclear respiratory factor-1 and 2; MyoD, myogenic determination factor; PGC-1 α, peroxisome proliferator-activated receptor gamma coactivator 1 α; young, participants 18–40 years old; middle aged, 40–65 years old; older, >65 years old; active, recreationally active; GPx, glutathione peroxidase; MnSOD, manganese superoxide dismutase; EcSOD, extracellular superoxide dismutase; MAPK, mitogen-activated protein kinase; DEM, diethyl maleate; SAPK, stress-activated protein kinase; IκBα, nuclear factor of kappa light polypeptide gene enhancer in β-cells inhibitor alpha*.

The discrepancy in findings are unclear, but likely include interstudy variations in the method and/or compounds used to modulate exercise-induced ROS, variations in the dose and treatment/supplementation time, and the often non-specific and/or ineffective action of antioxidant supplementation/treatment as a model for ROS inhibition (81, 191–195). Nevertheless, evidence provided so far supports a likely association between redox-sensitive SAPK signaling and skeletal muscle adaptation, specifically with that of mitochondrial biogenesis and endogenous antioxidant upregulation, which both participate in the regulation of glycemic control (6, 9).

## Positive and Negative Regulation of Glycemic Control by ROS

### Physical Inactivity, Excess Nutrient Intake, and Oxidative Stress

Chronic physical inactivity and overnutrition are associated with elevated systemic oxidative stress and the development of lifestyle disease in part through mitochondrial dysfunction (9). Metabolism of carbohydrate and lipids initiates the transfer of electrons from reducing equivalents (i.e., NADH, FADH_2_) into the mitochondrial electron transport system (ETS) (9). In the absence of energy demand, for example, physical inactivity, increased energy supply results in increased electron flow through the ETS and pumping of protons outside the mitochondrial membrane (9). When the membrane potential exceeds mitochondrial uncoupling capacity, electrons leak through complexes I and III reacting with O_2_ to form the free radical ${\text{O}}_{2}^{-}$, where it is catalyzed by MnSOD to H_2_O_2_ (196–199). Providing there is sufficient antioxidant activity, H_2_O_2_ is further reduced to H_2_O by antioxidants such as GSH and/or CAT (200). In pathological conditions in which antioxidant defense is insufficient, H_2_O_2_ can accumulate in the mitochondrial matrix and intermembrane space or diffuse outside the permeable mitochondrial outer membrane (201). Excess ROS production results in oxidative stress and the signaling events leading to insulin resistance and chronic metabolic disease (59). This proposed mechanism for physical inactivity and excess nutrient intake-induced chronic disease is supported by reports that mitochondrial-specific antioxidants, which attenuate mitochondrial ROS production, reverse high-fat diet-induced insulin resistance in rodents (198).

Elevated basal and/or postprandial hyperglycemia elicited through excess nutrient intake, physical inactivity, and insulin resistance also increases oxidative stress through the formation of advanced glycation end products (AGEs) (202). Activation of the AGE receptor stimulates ROS production through NADPH oxidase (203), opening of the mitochondrial permeability transition pore (204), and through suppression of enzymatic antioxidant defenses (205–207). Therefore, hyperglycemia has the potential to elicit a potentially deleterious redox environment conducive to insulin resistance.

Numerous studies have reported increased biomarkers of systemic oxidative stress in humans for up to 4 h after the ingestion of pure carbohydrate (208, 209), fat, and protein meals (210); mixed macronutrient meals high in fat (211–214) and high in carbohydrate (215); and high-fat liquid meals (216, 217). Larger meals and meals higher in lipid content elicit greater postprandial oxidative stress (218, 219). This has led to many studies researching the effects of high-fat meal ingestion on postprandial oxidative stress (211–214, 220); however, meals adhering to national recommended dietary guidelines also induce systemic postprandial oxidative stress (5).

A single bout of low to moderate-intensity exercise in healthy males can attenuate the postprandial oxidative stress response to a meal ingested 1–2 h before exercise (5, 216) and 24 h after exercise (215), in part through improved glucose and triglyceride processing and clearance and increased antioxidant activity (214). Acute high-intensity exercise may also attenuate postprandial oxidative stress (212, 213); however findings are inconsistent and likely depend on whether exercise is performed before or after meal consumption (5, 214).

The divergent effects of postexercise oxidative stress (physiological) and postprandial oxidative stress (pathological) on metabolic health may stem from the mechanisms of ROS production (59, 79). The pathological effects of oxidative stress are reported to primarily occur through mitochondrial dysfunction and excess mitochondrial ROS production (9), whereas exercise-induced ROS production are reported to primarily occur through alternative mechanisms such as NADPH oxidase and xanthine oxidase (95). Furthermore, the effects of ROS on glycemic control appear to occur on a spatiotemporal paradigm that involve the concentration of ROS (221), the exposure time of ROS (160), the type of ROS, organs and organelles involved (79), the subcellular localization of redox-sensitive protein signaling (160), and the type of exercise and postexercise recovery timepoint (14, 128, 222).

### Negative Regulation of Insulin Signaling by ROS

**Figure 5:**
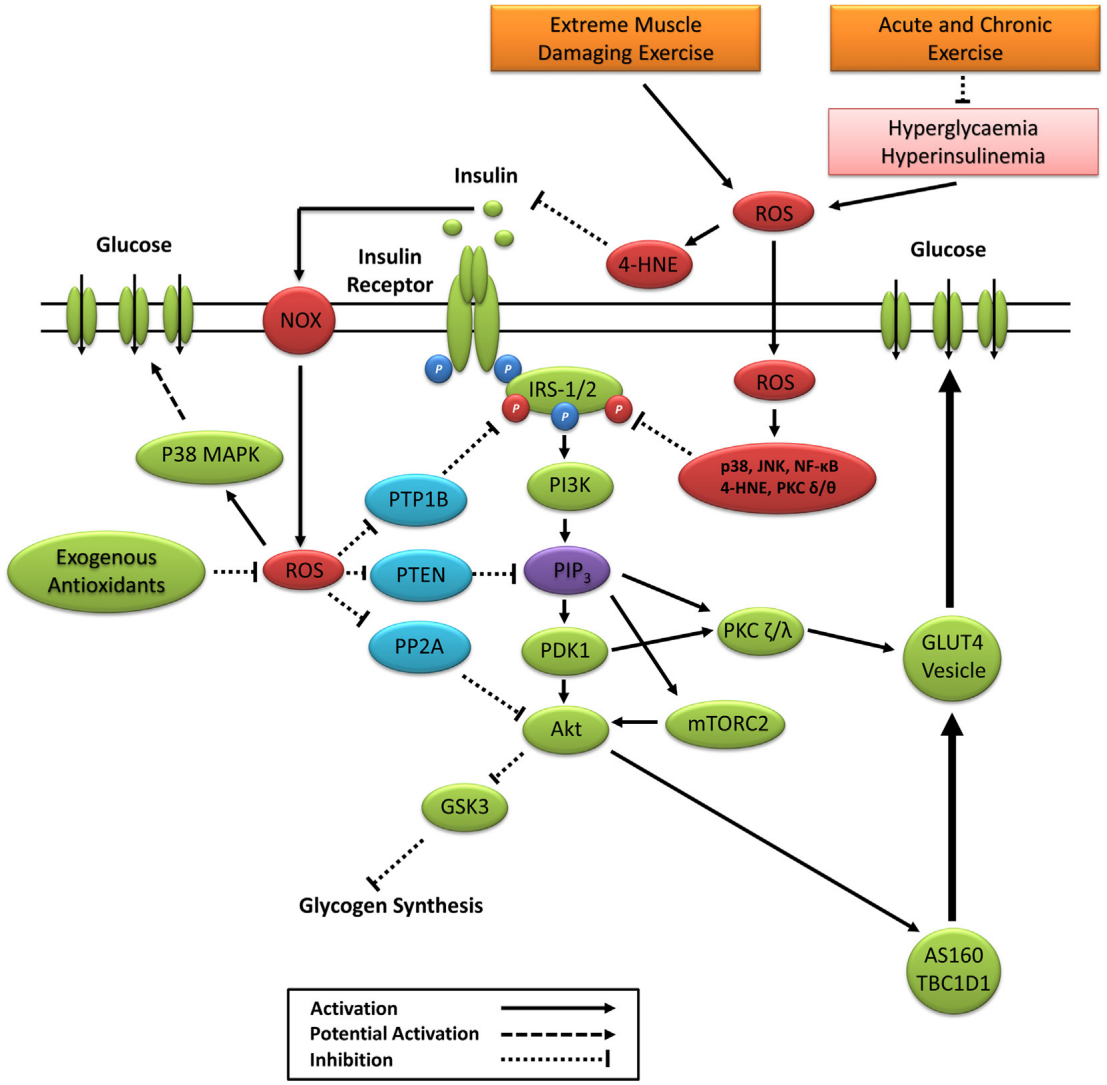
**Primary ROS signaling pathways involved in positive and negative regulation of insulin signaling**. 4-HNE, 4-hydroxynonenal; Akt, protein kinase B; AS160, Akt substrate of 160 kDa; GSK3, glycogen synthase kinase 3; IRS-1/2, insulin receptor substrates 1 and 2; JNK, c-Jun *N*-terminal kinases; mTORC2, mechanistic target of rapamycin complex 2; NF-κB, nuclear factor kappa-light-chain-enhancer of activated B cells; NOX, nicotinamide adenine dinucleotide phosphate oxidase; P38 MAPK, p38 mitogen-activated protein kinases; PDK1, phosphoinositide-dependent kinase-1; PI3K, phosphatidylinositol-3 kinase; PIP_3_, phosphatidylinositol (3,4,5)-trisphosphate; PKC, protein kinase C; PP2A, protein phosphatase 2; PTEN, phosphatase and tensin homolog; PTP1B, protein tyrosine phosphatase 1B; ROS, reactive oxygen species.

Sustained activation of redox-sensitive SAPK signaling pathways leads to impaired insulin signaling *via* increased phosphorylation of IRS-1 and IRS-2 on multiple serine and threonine residues, see the study by Copps and White (223) for a detailed review. Sustained IRS-1/2 serine phosphorylation impairs PI3K activity and downstream insulin signaling through attenuated tyrosine phosphorylation and IRS proteasomal degradation and subcellular relocalization (27, 160, 224–232) (Figure 5). The prevention of IRS-1 degradation through the inhibition of ROS and SAPK signaling restores insulin signaling and insulin-stimulated glucose uptake (8, 75, 181, 198, 233). Paradoxically, IRS serine phosphorylation may also be necessary for normal insulin signal transduction and glucose uptake (234). However, reports are contradictory (229, 231, 235) and depend largely on the length and degree of phosphorylation on specific serine residues (236). Previous studies have also reported that hyperinsulinemia initiates a negative feedback loop that inhibits insulin signaling and glucose uptake in part through SAPK-induced IRS serine phosphorylation (229, 231, 237–239).

### Positive Regulation of Insulin Signaling by ROS

The insulin receptor belongs to a subclass of the protein tyrosine kinase family. Positive regulation of the insulin signaling cascade is mediated in part through the oxidative inactivation of protein tyrosine phosphatases (PTP), which include protein tyrosine phosphatase 1B, phosphatase and tensin homolog, and protein phosphatase 2 (Figure 5). Insulin-induced inactivation of PTPs prevents the dephosphorylation of the insulin receptor (240), IRS-1 (241), and Akt proteins (242) and prevents the enzymatic degradation of PIP_3_ (243). The PTP superfamily signature motif contains an invariantly low-p*Ka* catalytic cysteine residue making it highly susceptible to reversible oxidation by ROS (244). ROS inactivation of PTP activity is associated with numerous cellular processes, including the regulation of cell proliferation, differentiation, survival, metabolism, and motility (244). Under basal conditions, antioxidant defenses such as CAT and peroxiredoxin create a reduced intracellular redox environment prioritizing PTP activity. Increased PTP activity suppresses kinase activity and maintains a dephosphorylated state of the IR, IRS-1, and inhibition of the PI3K/Akt signaling pathway (243, 245). The binding of insulin to the insulin receptor signals a burst of endogenous superoxide production, which is reduced to H_2_O_2_ creating a local oxidative redox environment (246–248). This oxidative redox environment favors the oxidation of catalytic cysteine to sulphenic acid, suppressing PTP activity and enhancing kinase activity and propagation of the insulin signaling cascade (9).

Insulin can elicit ROS production through enzymatic activation of NADPH oxidases (246–249). Furthermore, insulin-induced receptor tyrosine phosphorylation inactivates the endogenous membrane-associated antioxidant peroxiredoxin I, allowing for increased ROS production (78). Mahadev et al. (246) reported that NADPH oxidase-induced H_2_O_2_ enhances insulin signaling *via* oxidative inhibition of PTPs. Furthermore, palmitate-induced insulin resistance in rat skeletal muscle occurs through increased activity of PTPs *via* JNK and NF-κB (250), which is reversed 16 h after acute exercise in rats (222). Loh et al. (54) revealed that the elevated H_2_O_2_ response to insulin in GPx1^−/−^ mouse embryo fibroblasts coincided with elevated PI3K/Akt signaling, which can be suppressed by pretreating cells with ebselen, an NADPH oxidase inhibitor, or the antioxidant *N*-acetylcysteine. Subsequent experiments revealed that elevated H_2_O_2_ in GPx1^−/−^ mice increased PI3K/Akt signaling and glucose uptake through decreased PTP activity, which was attenuated by the ingestion of *n*-acetylcysteine (NAC) (54). Thus, redox-mediated PTP activity appears to be associated with both positive and negative regulations of insulin signaling and glucose uptake.

### Exercise-Induced ROS, SAPK Signaling, and Glycemic Control

Reactive oxygen species are readily induced through contraction of skeletal muscle (251–253). Importantly, contraction of skeletal muscle coincides with increased activation of redox-sensitive SAPK signaling pathways implicated in glucose metabolism (14, 160, 161, 254–256). Therefore, skeletal muscle SAPK signaling has emerged as a potential candidate for the postexercise enhancement of insulin sensitivity (14, 54, 182).

Loh et al. (54) reported that exercise-induced ROS in GPx1 knockout mice coincided with increased phosphorylation of Akt^(Ser473)^ and AS160^(Thr642)^ and enhanced insulin-stimulated glucose uptake 60 min after a single session of treadmill exercise. This beneficial effect on insulin sensitivity was reversed with NAC supplementation, suggesting that redox signaling is not only an important regulator of basal insulin signaling and glucose uptake but also postexercise enhancement of insulin sensitivity. Importantly, GPx1 knockout mice showed similar improvements in insulin sensitivity when measured immediately after exercise, supporting a growing consensus that the effects of postexercise-induced ROS on glycemic control are temporal (14, 222).

One of the first studies to indicate a regulatory role of redox signaling in exercise-induced enhancement of insulin sensitivity in humans was conducted by Ristow et al. (13). It was reported that vitamin C and E supplementation in humans attenuated the 4-week training-induced improvements in insulin sensitivity and gene expression of PGC-1α/β, SOD, GPx1, and CAT (13). Not all studies in humans and rodents have reported impaired exercise-induced improvements in insulin protein signaling and insulin sensitivity with antioxidant supplementation (146, 148, 257). Contradictory findings likely stem from variations in the type of antioxidant compound/s used, the dose used, the timing of supplementation, and the often non-specific and/or ineffective action of antioxidant supplementation for ROS inhibition in humans (81, 193, 194).

Enhanced glucose uptake approximately 4.5 h after one-legged knee extensor exercise in humans is reported to coincide with greater basal and insulin-stimulated p38 MAPK phosphorylation (182), highlighting SAPK signaling as a potential moderator of postexercise glucose metabolism. Trewin et al. (55) reported that NAC infusion attenuated whole-body insulin sensitivity approximately 5 h after exercise. Phosphorylation of p38 MAPK was lower immediately after exercise with NAC infusion; however, phosphorylation was not significantly different to baseline or the placebo after insulin stimulation. However, the null findings for p38 MAPK phosphorylation may be due to the timing of postexercise biopsies, the relatively small effect of NAC on insulin sensitivity (~6% reduction), and that NAC infusion was not maintained during the recovery period and subsequent insulin clamp (55). Interestingly, Parker et al. (14) demonstrated that a bout of high-intensity interval exercise prior to a 2-h euglycemic–hyperinsulinemic clamp in obese middle-aged males elicited greater insulin-stimulated p38 MAPK, JNK, NF-κB, and AS160^Ser588^ phosphorylation, which was associated with improved insulin sensitivity compared to a resting clamp. Equivocal findings in humans may stem from reports that postexercise skeletal muscle SAPK and insulin protein signaling are effected by training status and occur in an exercise intensity and postexercise time course-dependent manner (256, 258).

Berdichevsky et al. (160) reported similar JNK phosphorylation in C2C12 myoblasts and L6 myotubes treated with chronic oxidative stress (1 μM of H_2_O_2_ for 48 h) and acute oxidative stress conditions (500 μM of H_2_O_2_ for 3 h). Interestingly, chronic oxidative stress decreased insulin-stimulated Akt^(Ser473)^ phosphorylation, whereas acute oxidative stress enhanced insulin-stimulated Akt^(Ser473)^ and GSK3-α/β phosphorylation. Furthermore, acute oxidative stress exposure in insulin-resistant muscle cells rescues insulin-stimulated glucose uptake through increased IRS1 protein abundance; increased phosphorylation of JNK, Akt^(Ser473)^, Akt^(Thr308)^, and GSK3-α/β; and decreased IRS-1^(Ser307)^ phosphorylation (160). In contrast, Ropelle et al. (222) reported that a single bout of exercise in male rats reverses diet-induced insulin resistance 16 h later *via* attenuation of JNK, NF-κB, and IRS-1^(Ser307)^ signaling. It is possible that acute exercise enhances insulin signal transduction through the transient and immediate increase in ROS and SAPK signaling, which also leads to a delayed increase in antioxidant activity and subsequent attenuation of chronic oxidative stress and sustained SAPK signaling pathways associated with insulin resistance. Certainly, SOD protein content, SOD activity, and total antioxidant status increase and/or remain elevated for up to 16–24 h after exercise (116, 212, 259), whereas lipid-induced postprandial oxidative stress is attenuated (213).

**Figure 6:**
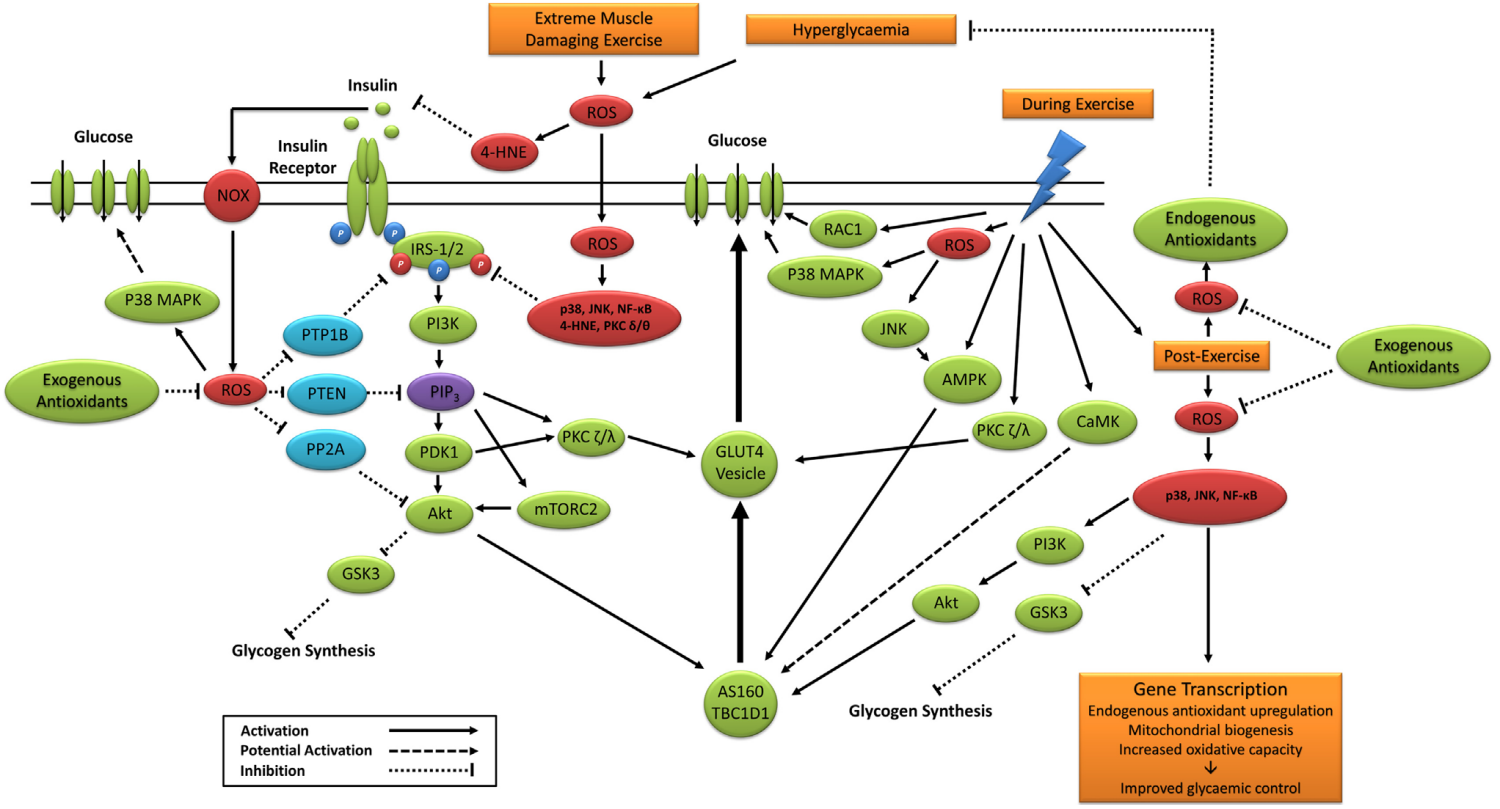
**Primary signaling pathways involved in contraction and insulin-stimulated glucose uptake and the potential role of ROS**. 4-HNE, 4-hydroxynonenal; Akt, protein kinase B; AMPK, 5′ adenosine monophosphate-activated protein kinase; AS160, Akt substrate of 160 kDa; CaMK, Ca^2+^/calmodulin-dependent protein kinase; GLUT4, glucose transporter 4; GSK3, glycogen synthase kinase 3; IRS-1/2, insulin receptor substrates 1 and 2; JNK, c-Jun *N*-terminal kinases; mTORC2, mechanistic target of rapamycin complex 2; NF-κB, nuclear factor kappa-light-chain-enhancer of activated B cells; NOX, nicotinamide adenine dinucleotide phosphate oxidase; P38 MAPK, p38 mitogen-activated protein kinases; PDK1, phosphoinositide-dependent kinase-1; PI3K, phosphatidylinositol-3 kinase; PIP_3_, phosphatidylinositol (3,4,5)-trisphosphate; PKC, protein kinase C; PP2A, protein phosphatase 2; PTEN, phosphatase and tensin homolog; PTP1B, protein tyrosine phosphatase 1B; RAC1, ras-related C3 botulinum toxin substrate 1; ROS, reactive oxygen species.

Taken together, previous studies support a potential role for exercise-induced redox-sensitive protein signaling and glycemic control (Table 2); however, specific mechanisms remain to be elucidated (Figure 6).

**Table 2:** **Summary of key findings from research investigating exercise, redox state, and enhancement of glycemic control**.

Reference	Participants/animals/cells^a^	Exercise stimulus	Redox manipulation	Time point/conditions	SAPK signaling	Glycemic control
Berdichevsky et al. (160)	Myocytes, myoblasts, and/or myotubes	Acute oxidative stress (simulated exercise)	Chronic oxidative stress Acute oxidative stress	Chronic oxidative stress Acute oxidative stress Insulin-resistant cells Insulin-resistant cells + acute oxidative stress	↑ p-JNK ↑ p-JNK ↑ p-JNK Greater ↑ p-JNK	↓ p-Akt^Ser473,Thr308^. ↓ glucose uptake ↑ p-Akt^Ser473,Thr308^ and ↑ p-GSK3β. ↑ glucose uptake ↑ p-IRS-1^Ser312^, ↓ IRS-1 and glucose uptake ↓ p-IRS-1^Ser312^, ↑ IRS-1 and glucose uptake

Castorena et al. (51)	Low-fat and high-fat diet fed rats	Swimming exercise		3 h postex. + Ins.	NC p-JNK	NC Akt^Ser473,Thr308^, IR^Tyr1162/1163^, IRS-1-PI3K ↑ pAS160^Thr642,Ser588^ ↑ Insulin sensitivity Greater ↑ in insulin sensitivity and pAS160^Thr642,Ser588^ in low-fat diet fed rats

Geiger et al. (233)	Male Wistar rats	*In vitro* contraction	p-p38MAPK inhibition	3 h postcontraction 3 h postcontraction + P38 MAPK inhibition Post-Ins. 3 h postcontraction + Ins. 3 h postcontraction + Ins. + p38MAPK inhibition	↑ p-p38 MAPK NC p-p38 MAPK Not measured Not measured Not measured	Not measured Not measured ↑ glucose uptake Greater ↑ glucose uptake Similar glucose uptake as previous condition

Higashida et al. (146)	Male Wistar rats	3-day swimming program or 3 weeks, 6 days/week swimming program	Vehicle or vitamin C + E supplementation	Post 3-day training Post 3-week training		3-day and 3-week training: vitamin C + E: similar ↑ in measures of mitochondrial protein content, ↑ GLUT4, ↑ glucose uptake

Loh et al. (54)	10 wild-type mice9 GPx1^−/−^ mice	Treadmill exercise	GPx1^−/−^ and *N*–acetylcysteine	Postex. + Ins. 1 h postex. + Ins.		GPx1^−/−^ mice: similar insulin sensitivity GPx1^−/−^ mice: greater ↑ p-Akt^Ser473^ and insulin sensitivity *N*-acetyl.cysteine: attenuated insulin sensitivity

Parker et al. (256)	8 young adults Active Healthy	Crossover design Cycling exercise: SIE: 4 × 30 s all-out sprints; 4.5-min recovery periods HIIE: 5 × 4-min cycling bouts at 75% of *W*_max_; 1-min recovery periods CMIE: 30 min at 50% of *W*_max_		Postex. 3 h postex.	All ex.: similar ↑ p-JNK, ↑ p-p38 MAPK, ↓ IκBα; SIE: ↑ p-NF-κB; CMIE and HIIE: NC p-NF-κB All ex.: similar ↑ p-JNK, ↑ p-p38 MAPK, ↓ IκBα. NC p-NF-κB	All ex.: NC IRS-1, similar ↓ p-Akt^Ser473^; SIE: ↑ p-IRS1^Ser307^, greatest ↓ p-AS160^Ser588^; HIIE: greatest ↑ p-IRS1^Ser307^, ↓ p-AS160^Ser588^; CMIE: ↑ p-IRS1^Ser307^, NC p-AS160^Ser588^ All ex.: NC IRS-1, similar ↓ p-Akt^Ser473^; SIE: NC p-IRS1^Ser307^, NC p-AS160^Ser588^; HIIE: NC p-IRS1^Ser307^, ↓ p-AS160^Ser588^; CMIE: ↑ p-IRS1^Ser307^, ↓ p-AS160^Ser588^

Parker et al. (14)	11 middle-aged males Sedentary Obese	Cycling exercise: HIIE: (4 × 4 min at 95% HRpeak; 2-min recovery periods)		Rest trial: 2 h post-Ins. Exercise trial: 1 h postex. 3 h postex + Ins.	↑ p-JNK, ↑ p-p38 MAPK, ↓ IκBα ↑ p-JNK, ↑ p-NF-κB, ↑ p-p38 MAPK, ↓ IκBα Greater ↑ p-JNK, greater ↑ p-NF-κB, greater ↑ p-p38 MAPK Similar ↓ IκBα	↑ p-IRS1^Ser307^, ↑ p-AS160^Ser588, Ser318^ ↑ p-AS160^Ser588^ Greater ↑ insulin sensitivity and greater ↑ p-AS160^Ser588^ Similar ↑ p-IRS1^Ser307^ and ↑ p-AS160^Ser318^

Picklo and Thyfault (257)	56 high-fat diet-induced obese Sprague-Dawley rats	Motorized wheel exercise. 12 weeks, 5 times per week	With and without vitamin C + E supplementation	Posttraining		Vitamin C + E: similar improvement in HOMA-IR and OGTT Attenuated measures of mitochondrial protein content

Ristow et al. (13)	40 young males (20 trained and 20 active) Healthy	Biking, running and circuit training. 4 weeks, 5 times per week	Placebo or vitamin C + E supplementation	Posttraining		Vitamin C + E: attenuated ↑ insulin sensitivity, mRNA expression of PPARγ, PGC-1α/β

Ropelle et al. (222)	Male Wistar rats Control (*n* = 6) Obese (*n* = 8) Obese + Ex. (*n* = 8)	Swimming exercise		16 h postex. + Ins.	Compared to control: obese: ↑ p-JNK, ↓ IκBα Compared to obese: obese + Ex.: ↓ p-JNK, ↑IκBα	Compared to both control and obese + Ex.: obese attenuated insulin sensitivity, PI3K, p-IRS-1/2, p-IR. ↑ PTP1B content/activity and p-IRS^Ser312^

Somwar et al. (181)	Male Wistar rats	*In vitro* contraction	p-p38 MAPK inhibition	Post-Ins. Post-Ins. + p38 MAPK inhibition Postcontraction Postcontraction + p38 MAPK inhibition	↑ p-p38 MAPK and activity Attenuated p38 MAPK activity ↑ p-p38 MAPK and activity Attenuated p38 MAPK activity	Not measured Attenuated glucose uptake Not measured Attenuated glucose uptake

Thong et al. (182)	7 young males Active Healthy	60 min of one-legged knee extension		3 h postex. 3 h postex. + 30 min Ins. 3 h postex + 100 min Ins.	↑ p-p38 MAPK Greater ↑ p-p38 MAPK p-p38 MAPK similar to previous time point	Not measured ↑ insulin sensitivity compared to control leg ↑ insulin sensitivity compared to control leg

Trewin et al. (55)	7 young adults Active Healthy	55 min cycling at 65% VO_2peak_ followed by 5 min at 85% VO_2peak_	Crossover: *N*–acetylcysteine and saline infusion	Postex. 3 h postex. 3 h postex. + 2 h Ins.	NAC: ↓ p-p38 MAPK Both: NC p-p38 MAPK Both: NC p-p38 MAPK	Both: NC p-Akt^Thr308,Ser473^, ↑ p-PAS160 Both: ↑ PAS160. NC p-Akt^Thr308,Ser473^ Both: ↑ p-Akt^Thr308,Ser473^, ↑ p-PAS160 NAC: ↑ insulin sensitivity

Yfanti et al. (148)	21 young males Active Healthy	Intense endurance training program 5 times/week for 12 weeks	Placebo or vitamin C and E supplementation	Posttraining		Vitamin C + E: similar ↑ insulin sensitivity, ↑ Akt, ↑ HXK2, ↑ GLUT4

*^a^Where appropriate sample sizes for animal research are reported*.

*Ins., insulin stimulation; NC, no change compared to baseline or control; ↑, significant increase compared to baseline or control; ↓, significant decrease compared to baseline or control; Akt, protein kinase B; AS160, Akt substrate of 160 kDa; CMIE, continuous moderate-intensity exercise; GLUT4, glucose transporter type 4; GSK3, glycogen synthase kinase 3; HIIE, high-intensity interval exercise; HOMA-IR, homeostatic model assessment for insulin resistance; HXK2, hexokinase II; IRS-1/2, insulin receptor substrates 1 and 2; JNK, c-Jun N-terminal kinases; NAC, n-acetylcysteine; NF-κB, nuclear factor kappa-light-chain-enhancer of activated B cells; P38 MAPK, p38 mitogen-activated protein kinases; PI3K, phosphatidylinositol-3 kinase; PPARγ/α/β, peroxisome-proliferator-activated receptor gamma/alpha/beta; PTP1B, protein tyrosine phosphatase 1B; SIE, sprint interval exercise; SAPK, stress-activated protein kinase*.

### Potential Mechanisms Linking SAPK Signaling and Enhancement of Glycemic Control

Modulation of glycogen synthesis by oxidative stress-induced SAPK signaling has been associated with glucose metabolism and regulation (27, 160, 182, 260). Transient stimulation of C2C12 myoblasts with H_2_O_2_ increases JNK, Akt, and GSK3α/β phosphorylation (160), suggesting the short exposure to exercise-induced ROS may increase postexercise glycogen synthesis and skeletal muscle glucose uptake. Likewise, postexercise enhancement of insulin-stimulated p38 MAPK phosphorylation is associated with postexercise glycogen depletion (182). Chan et al. (261) established that low intramuscular glycogen was associated with greater phosphorylation of nuclear p38 MAPK after 60 min of cycle exercise. In contrast, insulin stimulation of rat skeletal muscle exposed to 1 h of H_2_O_2_ (~90 μM) exhibits impaired insulin protein signaling, glycogen synthesis, and glucose uptake, despite increased p38 MAPK phosphorylation (27). Diamond-Stanic et al. (260) reported similar findings and proposed that p38 MAPK and GSK3 were unlikely to play a beneficial role in insulin-stimulated glucose uptake. Activation of JNK in skeletal muscle of mice is also associated with increased insulin-stimulated glycogen synthesis *via* the RSK3/GSK3 signaling pathway (262); however, greater postexercise JNK phosphorylation and insulin sensitivity in human skeletal muscle do not coincide with greater insulin-stimulated phosphorylation of GSK3 α/β (14).

Other potential pathways include JNK-, NF-κB-, and p38 MAPK-stimulated secretion of the recently identified insulin-sensitizing interleukin-6 (IL-6) (255, 263–265). Carey et al. (264) reported that IL-6 infusion increases insulin-stimulated glucose uptake in humans. Furthermore, IL-6 treatment in L6 myotubes coincides with increased glucose uptake and GLUT4 translocation, likely through AMPK pathways independent of the canonical insulin signaling cascade (264). Importantly, IL-6 secretion is increased following muscular contraction, likely *via* activation of JNK, NF-κB, and/or p38MAPK (261, 266, 267). It has also been reported that p38 MAPK inhibiters, alongside expression of a dominant-negative p38 mutant, impairs insulin-stimulated glucose uptake without reductions in GLUT4 translocation (254). Researchers concluded that p38 MAPK may exert its insulin-sensitizing effect through increased activation of translocated GLUT4 (254), but not all findings are supportive (268) and have yet to be investigated in humans. The reported subcellular redistribution of phosphorylated JNK from the cytoplasm to the nucleus with acute hydrogen peroxide exposure in skeletal muscle cells highlights another potential mechanism for the postexercise insulin sensitizing effect of JNK (160). Future research is warranted to explore the subcellular localization and activation of SAPK proteins after exercise and insulin stimulation in humans.

## The Future of Exercise-Induced Oxidative Stress, ROS, and Redox Signaling

Early studies, and the majority of current findings, rely primarily on associations and the assumption that increased/decreased ROS and/or markers of oxidative stress are reflective of, or are likely to lead to, increased/decreased redox signaling (91). Certainly, studies inhibiting or increasing ROS have been useful for establishing a relationship between ROS and certain biological outcomes such as glycemic control and exercise adaption (13, 14, 55, 135, 161). However, in the absence of specific redox signaling measurements such as protein cysteine oxidation or S-nitrosylation (162, 269), research studies are limited in their capacity to elucidate specific redox cellular signaling networks that are complex, compartmentalized, and spatiotemporally regulated (195). Future studies utilizing modern redox proteomics are required to establish the reversible and, in some cases, irreversible, redox regulation of kinases, phosphatases, transcription factors, and coactivators, thus establishing the “true” redox signaling role of exercise-induced ROS (195, 270–274). Furthermore, not all ROS are equal in their capacity to exert signaling effects (56). Future studies investigating exercise-induced oxidative stress should therefore strive to identify the specific ROS involved, which can be achieved through the use of robust techniques such as electron spin resonance, targeted fluorescent probes, and mass spectrometry (252, 275–278).

Despite their non-specificity and/or inability to adequately reflect redox signaling, the measurement of ROS, oxidative stress, and/or antioxidant activity in a biological sample provides insight into the effects of an intervention (e.g., exercise) on redox homeostasis and remains a useful biomarker of overall health and disease (91). As such, a combination of both traditional measures of redox biomarkers, the direct measurement of ROS, redox-sensitive protein signaling, and specific redox proteomics will likely provide a robust investigation of exercise-induced ROS and subsequent redox signaling.

## Conclusion

Physical inactivity, excess energy consumption, and obesity are associated with elevated ROS production, systemic oxidative stress, and sustained activation of redox-sensitive protein signaling pathways. If left unchecked, this chronic state of physiological stress can lead to insulin resistance, which likely contributes toward the development of cardiometabolic disease. Paradoxically, a single session of exercise transiently increases ROS, oxidative stress, and redox-sensitive protein signaling, yet both acute and regular exercises elicit favorable improvements in glycemic control and skeletal muscle adaptation. It appears that exercise-induced redox-sensitive protein signaling is necessary for adaptation to physiological stress. However, the spatiotemporal interplay between physical activity/inactivity, ROS, PTP activity, SAPK and MAPK signaling, insulin protein signaling, and the subsequent effects on glycemic control and cardiometabolic health remain unclear. Future research would benefit by employing a combination of human primary cell culture, animal research, modern proteomics, and immunohistochemistry/subcellular analysis of human tissue to elucidate the physiological relevance of transient oxidative stress (exercise induced), chronic oxidative stress (physical inactivity/excess nutrition intake), and the role of redox-sensitive protein signaling in human health and disease.

## Author Contributions

LP, CS, NS, and IL contributed to the conceptualization and overall design of the manuscript. LP drafted the initial version of the manuscript and figures. CS, NS, and IL critically revised the manuscript. All authors approved the final version of the manuscript.

## Conflict of Interest Statement

The authors declare that the research was conducted in the absence of any commercial or financial relationships that could be construed as a potential conflict of interest.
